# Immunotherapy in breast cancer: an overview of current strategies and perspectives

**DOI:** 10.1038/s41523-023-00508-3

**Published:** 2023-02-13

**Authors:** Véronique Debien, Alex De Caluwé, Xiaoxiao Wang, Martine Piccart-Gebhart, Vincent K. Tuohy, Emanuela Romano, Laurence Buisseret

**Affiliations:** 1 Academic Trials Promoting Team, Université Libre de Bruxelles (ULB), Hôpital Universitaire de Bruxelles (HUB), Institut Jules Bordet, Brussels, Belgium; 2 Radiotherapy Department, Institut Jules Bordet, Hôpital Universitaire de Bruxelles (HUB), Université Libre de Bruxelles (ULB), Brussels, Belgium; 3 Breast Cancer Translational Research Laboratory J.-C. Heuson, Institut Jules Bordet, Hôpital Universitaire de Bruxelles (HUB), Université Libre de Bruxelles (ULB), Brussels, Belgium; 4 Institut Jules Bordet, Hôpital Universitaire de Bruxelles (HUB), Université Libre de Bruxelles (ULB), Brussels, Belgium; 5 Department of Inflammation and Immunity, Lerner Research Institute, Cleveland Clinic, and Department of Molecular Medicine, Cleveland Clinic Lerner College of Medicine of CWRU, Cleveland, OH USA; 6 Centre for Cancer Immunotherapy, Medical Oncology Department, INSERM U932, Institut Curie, PSL Research University, Paris, France; 7 Medical Oncology Department, Institut Jules Bordet, Hôpital Universitaire de Bruxelles (HUB), Université Libre de Bruxelles (ULB), Brussels, Belgium

**Keywords:** Breast cancer, Breast cancer

## Abstract

Recent progress in immunobiology has led the way to successful host immunity enhancement against breast cancer. In triple-negative breast cancer, the combination of cancer immunotherapy based on PD-1/PD-L1 immune checkpoint inhibitors with chemotherapy was effective both in advanced and early setting phase 3 clinical trials. These encouraging results lead to the first approvals of immune checkpoint inhibitors in triple-negative breast cancer and thus offer new therapeutic possibilities in aggressive tumors and hard-to-treat populations. Furthermore, several ongoing trials are investigating combining immunotherapies involving immune checkpoint inhibitors with conventional therapies and as well as with other immunotherapeutic strategies such as cancer vaccines, CAR-T cells, bispecific antibodies, and oncolytic viruses in all breast cancer subtypes. This review provides an overview of immunotherapies currently under clinical development and updated key results from clinical trials. Finally, we discuss the challenges to the successful implementation of immune treatment in managing breast cancer and their implications for the design of future clinical trials.

## Introduction

Cancer immunotherapy represents one of the most significant advances in oncology in recent years. It has demonstrated impressive anti-tumor activity and a durable clinical benefit in diverse malignancies with recent success in triple-negative breast cancer (TNBC). Historically considered poorly immunogenic, breast cancer (BC) was initially not extensively investigated for its susceptibility to immunotherapy. However, recent breakthroughs with immune checkpoint inhibitors (ICI) in other cancers coupled with increasing evidence of the influence of the immune system in cancer behavior, have led to the development of clinical trials evaluating different types of immune therapeutic strategies for BC patients. The presence of tumor-infiltrating lymphocytes (TILs) in the tumor microenvironment (TME) reflects a pre-existing anti-tumor immune response and is associated with a better prognosis and response to chemotherapy^1^. The immune response captured through immune-related tumor gene expression in microarray-based analyses also demonstrated that immune gene signatures were associated with a favorable clinical outcome, particularly in TNBC and Human Epidermal Growth factor Receptor 2 (HER2)-positive BC^2,3^. In using immunophenotyping analyses or transcriptomic approaches, different immune cell subsets were identified in the TME and their participation in a pro- or anti-tumor immune response has been demonstrated given their influence on BC clinical outcomes^4^. Among CD8+ T cells, the cytotoxic subpopulation is able to kill cancer cells and is associated with improved survival in patients, whereas the presence of immunosuppressive regulatory CD4+ T cells (Tregs) or macrophages is associated with a worse prognosis^4^.

The extent and composition of immune infiltrates are highly variable between BC subtypes and within each subtype^5,6^. Therefore, it is expected that not all BC patients would benefit from the same immunotherapeutic strategy to restore or elicit an anti-tumor immune response^5^. Predictive biomarkers are required to select patients and tailor therapies beyond the established BC subtypes. Programmed death-ligand 1 (PD-L1) immunohistochemistry (IHC) expression is the most widely used biomarker, but not sufficient, as it only appears to have predictive value in metastatic TNBC (mTNBC). Tumor mutational burden (TMB) is a marker of tumor foreignness and immunogenicity, as mutated antigens are recognized by T cells to initiate a cytotoxic response. Mutational load is highly variable in BC, and tumors that present high TMB may respond more favorably to ICI^7^. Tumor antigens have also been investigated in vaccination strategies, as demonstrated by the increasing number of clinical trials evaluating the preventive and therapeutic effects of cancer vaccines. Emerging modalities such as bispecific antibodies (BsAbs) or adoptive cell therapies involving TILs or chimeric antigen receptor T (CAR-T) cells are an area of current research.

This review describes recent advances in immunotherapy to treat BC and summarizes the challenges of implementing such treatments in a heterogeneous disease. We also present a comprehensive overview of the immunotherapeutic combinations currently investigated in clinical trials.

## Clinical landscape and update of early results

**Fig. 1:**
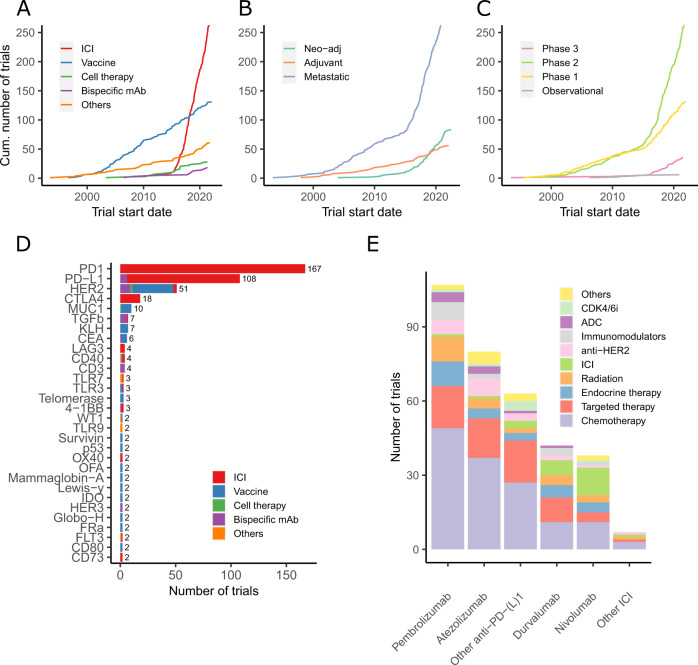
Immunotherapy trial landscape in breast cancer. Panels **A**–**C** show the number of clinical trials in breast cancer since early 2000, by immunotherapeutic approach (**A**), by trial setting (**B**), and by trial phase (**C**). Panel **D** shows the major immune targets. Only targets present in two or more trials are represented. The complete list of targets is available in online Supplementary Table 1. Panel **E** shows the histogram of combination trials with PD-1/PD-L1 ICI backbone. ADC antibody-drug conjugates, ICI immune checkpoint inhibitors, mAbs monoclonal antibodies, Neo-adj neoadjuvant.

The clinical development of immunotherapy in BC started more than 20 years ago, but it is only with the discovery of ICI that number of clinical trials testing immunotherapeutic strategies increased (Fig. 1A)^8^. In January 2022, 745 immunotherapy-based trials enrolling patients with solid tumors, including BC, were identified on clinicaltrials.gov, with 450 (60.4%) exclusively dedicated to BC. Interestingly, our analysis shows a constant increase in the development of vaccines in the last 20 years, whereas more recent immunotherapeutic approaches increased exponentially since 2015 (Fig. 1A).

The number of trials is increasing both in the advanced setting and in early BC. In 2018, the number of neoadjuvant trials exceeded the number of adjuvant trials (Fig. 1B), and a shift of phase 1 trials towards phase 2 and 3 trials is clearly observed (Fig. 1C). Of note, the large phase 3 trials are sponsored by pharmaceutical companies, whereas the observed rise of phase 2 investigator-initiated studies indicates an enhanced global effort to investigate novel immunotherapy strategies.

The most studied co-inhibitory receptor is programmed death-1 (PD-1). Multiple monoclonal antibodies (mAbs) targeting PD-1 or its ligand PD-L1 have been developed (Fig. 1D). Other molecules targeting immune checkpoints to prevent the inhibition of T cells (e.g., CTLA-4, LAG3, and TIGIT) or to stimulate T cells and increase their cytotoxic activity (e.g., OX-40 and 4-1BB) are being tested. HER2 represents the most studied target for vaccines but is also used by BsAbs and other directed therapies (Fig. 1D). Recently, new combination strategies beyond ICI aiming to increase response rates (RR) and clinical benefit have been initiated with the hope of improving survival outcomes (Fig. 1E).

## Immune checkpoint combinations

### Metastatic breast cancer

In early phase trials, PD-1/PD-L1 ICI was primarily evaluated in monotherapy, enrolling heavily pretreated metastatic patients^9^. The response rates (RR) were only 5–20%, with increased efficacy in patients with PD-L1-positive TNBC, lower tumor burden, and non-visceral disease^10^. Nevertheless, few responders achieved long-lasting responses with survival benefit^11,12^. However, the KEYNOTE-119 trial, in which pembrolizumab monotherapy was compared to chemotherapy, failed to improve overall survival (OS) beyond the first line in mTNBC (Table 1)^13^.

**Table 1:** The main results from principal phase 2 and 3 trials in metastatic and early breast cancer.

Trial	Study design	Setting	Number of subjects	Drug	Primary endpoint	Main results	Additional information	FDA approval
**IMpassion130**^16^	Phase 3 randomized controlled	mTNBC first line	902	**Arm A**: Nab-Paclitaxel + Atezolizumab **Arm B**: Nab-Paclitaxel + placebo	PFS and OS in ITT and PD-L1 + (hierarchical)	PFS: 7.2 vs 5.5mo HR = 0.80 (0.69-0.92) OS: 21.0 vs 18.7mo HR = 0.87 (0.725-1.02) PD-L1+: OS 25.4 vs 19.7mo HR = 0.69 (0.54-0.88) PFS: 7.5 vs 5.3 mo HR = 0.63 (0.50-0.80)	Testing in PD-L1+ population was not planned initially	**Withdrawn**
**KEYNOTE-355**^19,20^	Phase 3 randomized controlled	mTNBC first line	847	**Arm A**: Nab-Paclitaxel/Paclitaxel/Gemcitabine-Carboplatin + pembrolizumab **Arm B**: Nab-Paclitaxel/Paclitaxel/Gemcitabine-Carboplatin + placebo	PFS and OS in PD-L1 CPS score ≥10, ≥1, and ITT (hierarchical)	PFS: 7.5 vs 5.6mo HR = 0.82 (0.69-0.97) CPS ≥10: PFS: 9.7 vs 5.6mo HR = 0.66 (0.50-0.88) OS: 23 vs 16.1mo HR = 0.73 (0.55-0.95) CPS ≥1: PFS: 7.6 vs 5.6mo HR = 0.75 (0.62–0.91)	*p* value boundary for OS in CPS ≥1 not met, no ITT testing	**Yes**
**IMpassion131**^17^	Phase 3 randomized controlled	mTNBC first line	651	**Arm A**: Paclitaxel + Atezolizumab **Arm B**: Paclitaxel + placebo	PFS in PD-L1+ and ITT (hierarchical)	ITT: PFS: 5.7 vs 5.6mo HR = 0.86 (0.70-1.05)^a^ OS: 19.2 vs 22.8 mo HR = 1.12 (0.88–1.43) PD-L1+: PFS: 6.0 vs 5.7mo HR = 0.82 (0.60–1.12) OS: 22.1 vs 28.3 mo HR = 1.11 (0.76–1.64)	^a^ PFS in ITT population was not formally tested	No
**SAFIR02-BREAST IMMUNO**^93^	Phase 2	Metastatic HER2-negative 1st Line	199	**Arm A**: durvalumab **Arm B**: chemotherapy	PFS	mPFS: 2.7 vs 4.6mo HR = 1.4 (1.00–1.96), *p* = 0.047 mOS: 21.7 vs 17.9mo HR = 0.84 (0.54–1.29) *p* = 0.423 TNBC PD-L1+ (32) mOS = 27.3 vs 12.1mo HR = 0.37 (0.12–1.13) *p* = 0.0678	Ten patients had therapeutic break	No
**KEYNOTE-119**^13^	Phase 3 randomized open-label	mTNBC >1st Line	1098	**Arm A**: Pembrolizumab **Arm B**: Physician’s chemotherapy choice	OS in ITT and PD-L1+	OS: 9.9 vs 10.8 mo HR = 0.97 (0.82–1.15) PFS: 2.1 vs 3.3 mo HR = 1.60 (1.33–1.92) CPS >10: OS: 12.7 vs 11.6mo HR = 0.78 (0.57–1.06) PFS: 2.1 vs 4.3 mo HR = 1.14 (0.82–1.59)		No
**Topacio/KEYNOTE-162**^30^	Phase 2 open-label	mTNBC <third line	55	Niraparib + Pembrolizumab	ORR	ORR in full analysis population: 18% (90% CI 10–29) RR in *gBRCAmut*: 47% (90% CI 24–70)	DCR in full analysis 42% (90% CI 31–54)	No
**PANACEA**^26^	Phase 1b/2 open-label	Metastatic HER2-positive >first line	52	Trastuzumab + Pembrolizumab	OR in PD-L1+	In PD-L1+: OR: 6/40 patients (90% CI 7–29) PFS: 2.7mo (90% CI 2.6–4.0)	OS: PD-L1+: NR (13.1-NR) vs PD-L1-: 7mo (90% CI 4·9–9·8)	No
**KATE2**^27^	Phase 2 randomized, double-blind	Metastatic HER2-positive ≥first line	202	**Arm A**: T-DM1 + atezolizumab **Arm B**: T-DM1 + placebo	PFS in ITT	PFS: 8.2 vs 6.8 8mo HR = 0.82 (0.55–1.23) *p* = 0.33 In PD-L1 + : PFS: 8.5 vs 4.1mo HR+: HR: 1.08 (0.64–1.82) HR-: HR: 0.58 (0.31–1.10)	OS: HR = 0.74 (0.42–1.30)	No
**ENHANCE 1**^94^	Phase 2 randomized open-label	Metastatic Luminal >third line	88	**Arm A**: Eribulin + Pembrolizumab **Arm B**: Eribulin	PFS	PFS: 4.1 vs 4.2mo HR = 0.80 (0.50–1.26) *p* = 0.33	Cross-over 14 patients	No
**MEDIOLA**^a^^29^	Phase 1b/2 open-label	Metastatic HER2-negative >first line	34	Olaparib + Durvalumab	DCR	DCR at week 12: 80% (90% CI: 64.3–90.9) DCR at week 28: 50% (90% CI 33.9–66.1)	ORR at week 12 63.3% (95% CI 48.9–80.1)	No
**GELATO-trial**^95^	Phase 2	Metastatic HER2-negative Lobular <third line	40	Carboplatin + Atezolizumab	PFS at 6mo	4/23 patients free of PD at week 24 ORR: 19% CBR at week 24: 29%	First analysis	
**KEYNOTE-522**^34^	Phase 3 randomized controlled	Neoadjuvant Adjuvant TNBC	1774	**Arm A:** Carboplatin + Paclitaxel + 4xAC + pembrolizumab -> pembrolizumab in adjuvant **Arm B:** Carboplatin + Paclitaxel + 4xAC + placebo -> placebo in adjuvant	8y-EFS	pCR: 64.8 vs 51.2% *p* < 0.001 PD-L1+: pCR 68.9 vs 54.9% Events: 15.7 vs 23.8% HR = 0.63 (0.48–0.82)	Favorable trend in OS; Long-term FU awaited	**Yes**
**GeparNUEVO**^38^	Phase 2 randomized controlled	Neoadjuvant TNBC	174	**Arm A:** Durvalumab + nab-paclitaxel -> EC **Arm B**: Placebo + nab-paclitaxel -> EC	pCR	pCR: 53.4 vs 44.2% OR:1.45(0.80–2.63) 3y-iDFS 84.9 vs 76.9% HR = 0.54, (0.27–1.09), stratified log-rank *p* = 0.0559 3y-OS: 95.1 vs 83.1% HR = 0.26 (0.09–0.79) *p* = 0.0076 in PD-L1+**:** pCR 58 vs 50.7% *p* = 0.363	Higher pCR in high TILs and high TMB and WoO sTILs stratification for iDFS	No
**NeoTRIPaPDL1**^37^	Phase 3 randomized open-label	Neoadjuvant TNBC	280	**Arm A**: Carboplatin + Nab-Paclitaxel + atezolizumab -> adjuvant AC/EC **Arm B**: Carboplatin + Nab-Paclitaxel -> adjuvant AC/EC	5y-EFS	pCR: 48.6 vs 44.4% OR: 1.18 (0.74–1.89), *p* = 0.48 PD-L1+**:** pCR 51.9 vs 48% OR: 2.08 (1.64–2.65)	EFS data not mature TILs unbalanced	No
**IMpassion031**^35^	Phase 3 randomized controlled	Neoadjuvant TNBC	455	**Arm A**: Nab-paclitaxel + 4xAC+atezolizumab **Arm B**: Nab-paclitaxel + 4xAC + placebo	pCR in ITT and PD-L1+	pCR: 57.6 vs 41.1% *p* = 0.0044 PD-L1+**:** pCR 68.8 vs 49.3% *p* = 0.021	EFS data not mature	No
**GIADA**^41^	Phase 2	Neoadjuvant Luminal B	43	EC-> Nivolumab+ triptorelin + exemestane	pCR	pCR: 16.3% (7.4–34.9)	Any PD-L1	No
**I-SPY 2**^40^	Phase 2 randomized open-label	Neoadjuvant HER2-negative	181	**Arm A**: weekly paclitaxel followed by AC+ pembrolizumab **Arm B**: weekly paclitaxel followed by AC [other arms in I-SPY-program not mentioned here]	pCR	pCR rates: HER2-: 44 vs 17% HR+ and HER2-: 30 vs 13% TNBC: 60 vs 22%	Pembrolizumab was the first of 10 agents in the I-SPY program to graduate in the HR-positive/ERBB2-negative signature.	No

*AC* anthracycline-cyclophosphamide, *CBR* clinical benefit rate, *CPS* combined positive score, *DCR* disease control rate, *EC* epirubicin-cyclophosphomide, *EFS* event-free survival, *FU* follow-up, *HR* hazard ratio, *HR−* hormone-receptor negative, *HR+* hormone-receptor-positive, *ITT* intention-to-treat, *OR* overall response, *ORR* objective response rate, *OS* overall survival, *pCR* pathological complete response, *PD* progressive disease*, PD-L1* programmed death-ligand 1, *PFS* progression-free survival, *TILs* tumor-infiltrating lymphocytes, *TMB* tumor mutational burden, *TNBC* triple-negative breast cancer, *WoO* window-of-opportunity.

^a^Only breast cancer cohort.

Higher RR were observed with ICI combined with chemotherapy as first-line therapy in advanced TNBC, leading to randomized phase 3 trials in this setting^10,14^. The IMpassion130 trial demonstrated a gain of 2.5 months in progression-free survival (PFS) for patients treated with atezolizumab plus nab-paclitaxel whose tumors have PD-L1 ≥1% immune cells with the VENTANA SP142 immunohistochemistry (IHC) assay^15^. Based on these results, atezolizumab received accelerated approval from the United States Food and Drug Administration (FDA) in March 2019. However, FDA approval for atezolizumab was later withdrawn due to a lack of clinical benefit, because the final PFS and first OS interim analyses in the intention-to-treat (ITT) population did not cross the boundary for statistical significance^16^. The initially planned testing procedure was hierarchical, meaning that the analysis in the PD-L1 positive subgroup could be tested only if the primary endpoint in the overall cohort was met. Therefore, the OS results suggesting a survival benefit in the PD-L1 positive subgroup results must be interpreted with caution. Furthermore, the IMpassion131 trial enrolled a similar population but evaluated the combination of atezolizumab with paclitaxel (instead of nab-paclitaxel), and it also failed to demonstrate an improved outcome (neither PFS nor OS) even in the PD-L1-positive subgroup (Table 1)^17^. The use of immunosuppressive steroids for premedication to prevent hypersensitivity reactions with paclitaxel has been incriminated in these discordant results. In the ongoing IMpassion132 trial enrolling TNBC patients with early relapses (<12 months), the chemotherapy partners are carboplatin and gemcitabine or capecitabine^18^. In the KEYNOTE-355 trial, pembrolizumab was used in combination with paclitaxel, nab-paclitaxel, or gemcitabine plus carboplatin in first-line therapy for patients with mTNBC. The primary PFS results led to the approval of the drug by the FDA in November 2020 for patients with PD-L1-positive tumors^19^. Recently, the OS benefit was confirmed in patients with a PD-L1 combined positive score (CPS) ≥10 assessed by the IHC 22C3 pharmDx test^20^.

In luminal BC, the first attempts to combine ICI and chemotherapy were disappointing. In initial trials, no improved outcomes were reported, such as in a phase 2 study evaluating eribulin with or without pembrolizumab in metastatic luminal BC^21^. Results are expected from ongoing studies investigating the safety and efficiency of ICI in combination with endocrine therapies and Cyclin D Kinase 4/6 inhibitors (CDK4/6i). In preclinical models, CDK4/6i enhanced tumor antigen presentation, decreased Tregs proliferation, and modulated T cell activation by reducing the expression of inhibitory receptors such as PD-1^22,23^. The phase 1b trial, evaluating the combination of abemaciclib with pembrolizumab with or without endocrine therapy in ER-positive metastatic BC, with or without anastrozole, were complicated by increased hepatic toxicity, interstitial lung disease, and two toxic death in the triplet arm^24^. In contrast, the triple association of letrozole, palbociclib, and pembrolizumab was well tolerated in a phase 1/2 trial^25^.

In metastatic HER2-positive BC, the combination of trastuzumab with pembrolizumab showed a 15% RR in patients with trastuzumab-resistant PD-L1-positive tumors^26^. In combination with T-DM1, atezolizumab did not improve PFS but increased toxicity^27^.

Poly ADP ribose polymerase (PARP) inhibitors can lead to DNA damage and genomic instability, which could increase cancer cell immunogenicity and enhance the sensitivity to immunotherapies^28^. In BRCA-deficient BC, the combination of ICI with PARP inhibitors is under investigation. The RR (objective RR or disease control rate) was promising in two phases 2 trials evaluating the combination of durvalumab and olaparib or pembrolizumab and niraparib in first-line or pretreated patients with germline *BRCA1* or *BRCA2* mutations (Table 1)^29,30^.

### Early breast cancer

Although many questions remain unanswered in the metastatic setting, several trials examined the use of immunotherapy in early BC. In theory, the early setting could be more appropriate for immunotherapy as the tumor burden is more limited, the biological background is more homogeneous, and the TME is less immunosuppressive and unimpacted by previous systemic treatments^31^. The majority of trials in early BC are now conducted in a neoadjuvant rather than in an adjuvant setting (Fig. 1B) because it offers the advantage of evaluating the clinical and imaging response before surgery and the pathological response after surgery, the latter being a possible surrogate endpoint for the long-term clinical benefit^32^. Moreover, the presence of the primary tumor could serve as a source of neoantigens. Notably, in preclinical models, the neoadjuvant immunotherapeutic approach demonstrated enhanced efficacy compared with the adjuvant setting^33^.

Similarly, as with metastatic disease, the majority of neoadjuvant trials were conducted in the TNBC subtype. In the landmark phase 3 KEYNOTE-522 trial, stage II and III patients received neoadjuvant chemotherapy (NACT) associated with pembrolizumab or placebo concomitant with NACT and then continued in the adjuvant setting^34^. The pathological complete response (pCR) rates were superior in the experimental arm (64.8 vs. 51.2%), and the overall pCR benefit was more significant for patients with node-positive disease (∆ pCR rate of 20.6 vs. 6.3%) (Table 1). The estimated event-free survival (EFS) rate at 36 months favored the pembrolizumab-chemotherapy combination (HR = 0.63, 95% CI 0.48–0.82, absolute gain 7.7%)^34^. The combination of neoadjuvant pembrolizumab plus chemotherapy, followed by adjuvant pembrolizumab, is an FDA-approved regimen for early TNBC as of July 2021.

While the KEYNOTE-522 trial used paclitaxel with carboplatin followed by anthracycline with cyclophosphamide every 3 weeks, combined with an anti-PD-1, the neoadjuvant trials IMpassion031 and GeparNUEVO combined nab-paclitaxel with an anti-PD-L1 (atezolizumab or durvalumab)^35–37^. The NeoTRIPaPDL1 trial combined nab-paclitaxel with carboplatin without anthracyclines in the neoadjuvant setting^37^. In IMpassion031, the addition of atezolizumab to nab-paclitaxel followed by dose-dense anthracycline-based chemotherapy resulted in a significant increase in pCR rate: 41 vs. 58%, (∆ pCR rate 17%, 95% CI 6–27, one-side *p* = 0.0044) (Table 1)^35^. However, NeoTRIPaPDL1 and GeparNUEVO trials could not demonstrate a substantial increase in pCR rates, highlighting the complexity of comparing different trials^37,38^. Even if there had been no difference in pCR rates in the GeparNUEVO trial, the addition of durvalumab to NACT significantly improved 3-year disease-free survival (DFS) and OS, questioning the validity of pCR as a surrogate endpoint in neoadjuvant immunotherapy trials (Table 1)^38^. Interestingly, pCR was only improved in patients treated in the window-of-opportunity part, in which durvalumab was given for 2 weeks before starting chemotherapy. Contrarily to the metastatic setting, PD-L1 IHC expression was not predictive of pCR, while TIL levels and dynamic TILs increase were associated with a better response in the retrospective analyses of KEYNOTE-173, GeparNuevo, and NeoTRIPaPDL1 trials^7,37,39^.

Less data were available for luminal and HER2-positive BC^40–42^. In phase 2 adaptively randomized I-SPY2 trial, adding pembrolizumab to NACT (weekly paclitaxel followed by doxorubicin-cyclophosphamide) was shown to be beneficial amongst patients with HER2-negative BC^40^. Pembrolizumab increased the pCR rate from 13 to 30% in luminal BC, which is a notable result given that in the metastatic setting, no benefit of ICI was found in this subtype. Nevertheless, compared to TNBC, the chemotherapy-ICI combination seems to generate lower pCR rates in luminal cancer, as expected, given its ‘colder’ immune phenotype. The ongoing phase 3 KEYNOTE-756 trial will shed light on the possible benefit of adding ICI to chemotherapy in grade III luminal BC^42^. The use of priming agents to elicit an immune response might be necessary to turn cold luminal BC into hot tumors^43^. For example, radiation therapy, which is a DNA-damaging agent, can be used to induce T cell priming via antigenic release and MHC-I upregulation. In addition, radiation activates innate immunity through several mechanisms, such as dendritic cells (DCs) activation^44^. This strategy is under evaluation in the Neo-CheckRay trial in luminal B MammaPrint high-risk BC^45^. The neoadjuvant chemotherapy-free strategy with ICI combined with endocrine therapy and CDK4/6i for luminal early BC resulted in increased hepatic toxicity^46^.

In HER2-positive BC, the randomized placebo-controlled phase 3 study IMpassion050 that evaluated the addition of atezolizumab to NACT and dual anti-HER2 blockade did not induce a significant increase in pCR rate in ITT nor PD-L1 positive population^47^. In addition, the median EFS, a secondary endpoint, was not reached in both arms^48^.

Fewer studies are being conducted in the adjuvant and post-neoadjuvant settings (Fig. 1B). Indeed, larger sample sizes are required as well as a longer follow-up, therefore exposing more patients with potentially curable BC to a hypothetically effective and potentially toxic experimental treatment. Of note, the continuation of ICI after neoadjuvant chemotherapy is still unclear in the context of post-neoadjuvant therapies with capecitabine in TNBC and olaparib for patients with germline *BRCA1* or *BRCA2* mutations^49,50^.

Longer follow-up will help to better delineate the benefit versus harm ratio of ICI, which will ultimately dictate the optimal use of immunotherapeutic approaches in early BC. Although the safety profiles with ICI in BC clinical trials were comparable to clinical trials in other tumor types, the risk of long-term side effects in patients treated with curative intent should be taken into consideration as some immune-related adverse events (irAE) could be responsible for chronic diseases^51,52^. Moreover, some irAE should be carefully assessed in the perioperative period, particularly endocrine toxicity such as hypopituitarism with the potential risk of adrenal crisis during or after surgical intervention^51,53^.

## Breast cancer vaccines

When the FDA approved trastuzumab in 1998 as the first monoclonal antibody for cancer treatment, the entire approach to cancer therapy changed. Ever since, there has been a relentless focus on HER2 as a predominant therapeutic target for HER2-positive cancers. However, despite the effectiveness of HER2 as a target for antibody-mediated receptor antagonism, it has met with conflicting and often perplexing results as a cancer vaccine target.

HER2 is a large molecule; therefore, most of the human HER2 cancer vaccines target one or more of the following three HER2-derived peptides: (1) E75 (Nelipepimut-S, NP-S, HER2 369–377, or NeuVax), an HLA-A2-restricted non-peptide derived from the extracellular domain of HER2 and designed to activate CD8+ T cells; (2) GP2 (HER2 654–662), another HLA-A2-restricted nonapeptide derived from the transmembrane domain of HER2 and also designed to activate CD8+ T cells in an HLA-A2-restricted manner; and (3) AE37 (HER2 776–790) an MHC class-II restricted 12-mer peptide derived from the intracellular domain of HER2 but modified by the addition of the four amino acids long Ii-Key peptide LRMK for enhancing the activation of CD4+ T cells^54^.

The results of phase 1/2 trials involving vaccination of BC patients with one or more of these HER2 peptides showed no significant clinical benefit, but exploratory subgroup analyses surprisingly indicated that patients with HER2-low-expressing tumors, including TNBC patients, may have derived a clinical benefit^55,56^. However, a subsequent phase 3 clinical trial involving E75 vaccination of patients, including TNBC patients, with node-positive HER2-low expressing breast tumors was stopped early when an interim analysis of the trial data showed that there was no significant difference in the primary endpoint of DFS between E75 vaccinated and placebo vaccinated subjects^57^.

Despite the confounding use of a HER2 vaccine in patients with HER2-low and HER2-negative BC, treatment of mTNBC with AE37 peptide vaccination has continued (NSABP FB-14). Moreover, a dendritic cell vaccine targeting HER2 and HER3, has been used to treat TNBC patients with brain metastases^58^. Further confusing the area, a recent meta-analysis of 24 clinical studies involving a total of 1704 vaccinated patients and 1248 control subjects found that E75 vaccination caused significant improvement in disease recurrence rate and DFS but no significant difference in OS^59^. One can only speculate how a vaccine targeting HER2 could possibly be effective in treating patients with HER2-negative tumors but not HER2-positive tumors, yet the confounding saga of HER2 vaccination continues.

The HER2 vaccine story certainly reveals the frustration that clinical investigators have had in finding a targeted treatment for TNBC, a BC subtype that expresses none of the traditional targets for BC therapy, including estrogen and progesterone receptors, and HER2. Moreover, TNBCs overexpress several non-HER2 tumor-associated antigens (TAAs), many of which have been the focus of numerous cancer vaccine clinical trials.

Perhaps the most commonly targeted non-HER2 TAAs for cancer vaccination have been the cancer-testis antigens (CTAs). These proteins are normally expressed in embryonic stem cells and testicular germ cells, minimally expressed in most other normal tissues but often expressed at high levels in many different tumors^60^. Several hundred CTAs have been identified, and many have served as targets in vaccination involving patients with TNBC^61^. Perhaps the most notable is cancer/testis antigen 1B (NY-ESO-1)^62^. Several other CTAs have been targeted in the vaccination of TNBC patients, including Wilms’ tumor protein (WT1)^63,64^ the melanoma antigen gene protein-12 (MAGE-12), the folate receptor alpha (FRα), the T-box transcription factor brachyury^65^ and the tumor suppressor transcription factor p53^66^.

One of the more interesting TAAs for targeting TNBC is Mucin 1 (MUC1), a hyperglycosylated, immunologically unavailable protein in many normal epithelial cells but a hypoglycosylated, immunologically available protein in several malignant tumors, including TNBC^67^. Several MUC1 vaccines have been tested in TNBC clinical trials. A number of cancer vaccines that target multiple TAAs have been developed for therapy against TNBC, including the PVX-410 vaccine that consists of peptides derived from the transcription factor X-box binding protein 1 (XBP1), the plasma cell marker syndecan-1 (CD138), and the NK cell receptor CD319 (CS1), as well as STEMVAC, a DNA vaccine encoding multiple peptides of CD105 (Endoglin), Y-box binding protein 1 (Yb-1), SRY-box 2 (SOX2), cadherin 3 (CDH3), and murine double minute 2 (MDM2) proteins. In addition, the vaccine-based immunotherapy regimen-2 (VBIR-2) has been used to treat patients with non-small cell lung cancer (NSCLC) and patients with TNBC, and apparently consists of several immunomodulators as well as multiple vaccinations against prostate-specific antigen (PSA), prostate-specific membrane antigen (PSMA), and prostate stem cell antigen (PSCA). Vaccination against PSMA and the preferentially expressed antigen in melanoma (PRAME) has also been used to treat TNBC patients^68^.

It is important to note that not all TNBC vaccines target TAA proteins. Indeed, tumor-associated carbohydrate (TAC) antigens that are frequently poor immunogens can be targeted using molecular mimic peptides or mimotopes that induce antibodies that cross-react with the human TAC antigen^69^. Such a mimotope vaccine called P10s-PADRE is currently being tested in clinical stage I-III TNBC patients. In addition, a vaccine that targets a non-protein hexasaccharide with a ceramide attached to its terminal glucose ring, the Globo H glycosphingolipid antigen, has reached phase 3 clinical trial status in patients with Globo H+ TNBC tumors^70^.

Despite decades of intense efforts using therapeutic cancer vaccines, the results have been modest or confounding at best. However, much has been learned about immunology in the past several decades, and recent cancer vaccine strategies may prove to be more effective than prior generations of cancer vaccines. Individual tumors have their own set of distinct mutations, many of which have the potential to be highly immunogenic for each individual patient. Such mutated proteins are called neoantigens, and recent clinical trials have focused on isolating these neoantigens and vaccinating individual TNBC test subjects with personalized neoantigen vaccines that include traditional vaccine/adjuvant combinations, vaccination with DNA-based vaccines, vaccination involving autologous dendritic cells, and even mRNA vaccination.

Finally, in light of the very successful prophylactic childhood vaccination program against infectious diseases, one may wonder why TNBC cancer vaccines have long been exclusively treatment vehicles^71^. Even when vaccines are used to prevent the recurrence of pre-existing tumors, they are still treatment vehicles. However, it has recently been proposed that vaccination against the human lactation protein, α-lactalbumin, may provide safe and effective primary prevention of TNBC because α-lactalbumin is a “retired” self-protein that is expressed exclusively in the breast only during late pregnancy and lactation but is expressed in >70% of TNBCs^72^. Thus, preemptive α-lactalbumin immunity provided to women at high risk for developing TNBC due to carrying mutations in their *BRCA1* genes^73^ may provide safe and effective primary prevention of TNBC as long as lactation is avoided. A phase 1 clinical trial to start this clinical testing process has very recently been initiated, with the first patient vaccinated in 2021. Thus, perhaps the focus of cancer vaccinations in the future may be to provide therapeutic immunity in a personalized manner to multiple neoantigens or to provide neoantigen or ‘retired’ self-protein immunity preemptively for the greatest effectiveness.

## Other immunotherapeutic strategies under development

Adoptive cell therapies (ACTs) consist of identifying and isolating peripheral blood or tumor-resident T cells in order to modify, activate and expand these cells ex vivo before transferring them back into the patient^74^. ACTs can be classified into TIL-based therapies, T cell receptor (TCR) gene therapy, and CAR-T cells. The latter technology has already provided prolonged responses and remissions for patients with advanced hematological malignancies^75^.

First attempts to reintroduce autologous lymphokine-activated lymphocytes to treat patients with advanced solid tumors were undertaken years ago without relevant results in BC patients^76^. Of note, clinical trials evaluating ACTs were conducted in early phase trials enrolling a small number of patients, including very few with BC^77^. Recently, infusion of autologous activated lymphocytes against specific tumor antigens was demonstrated able to induce a long-lasting response in a patient with chemotherapy-refractory luminal metastatic BC treated with mutant-protein-specific TILs in conjunction with IL-2 and pembrolizumab^78^. In a study evaluating the feasibility of c-MET CAR-T cells, the best response was a stable disease for only one patient with ER-positive HER2-negative disease among the six patients with metastatic BC^79^. In solid tumors, the development of ACTs has been hampered by the heterogeneity of the antigenic landscape, the hostile TME conditions, and the lack of T cell infiltration in the tumor nests. Several strategies are under development to overcome these issues. Thus, promising CAR-T cell targets like HER2, MUC1, or Mesothelin have been identified for the treatment of BC patients^80^. The identification of neoantigens and the use of other immune cell types, such as NK cells or DCs offer new opportunities for ACTs.

Another challenge to develop ACTs is the toxicities related to lymphodepletion and to immune-mediated side effects such as neurotoxicity and cytokine release syndrome, two potentially lethal conditions. Cytokine release syndrome is a systemic inflammatory response with organ dysfunction that can be reversible if promptly diagnosed and managed^81^. In addition to the management of these toxicities, the complexity of manufacturing ACTs limits the development of cellular therapy programs in specialized cancer centers^82^.

Another type of engineered molecule are BsAbs designed to recognize two different epitopes or antigens on tumor cells and immune cells allowing immune recognition of these cancer cells^83^. A variety of BsAbs relevant to BC are in development^84^. Zanidatamab, BsAb, targets two different HER2 epitopes, in combination with chemotherapy, was well-tolerated, and has shown anti-tumor activity in heavily pretreated HER2-amplified metastatic BC patients^85^. In TNBC, BsAbs from a large panel of tissue agnostic targets such as CD3, CEACAM5, epithelial cell adhesion molecule (EpCAM), epithelial growth factor receptor (EGFR), mesothelin including Trop2 are under investigation^83^.

## Conclusions and perspectives

Although the development of cancer immunotherapy in BC began more than 20 years ago, its integration into patient care was slower than in other tumor types. The current extensive clinical research landscape will hopefully change this situation and expand the use of ICI and other immunotherapies in BC beyond the TNBC subtype. As reviewed herein, the number of clinical trials evaluating multiple immunotherapeutic strategies is increasing across all BC subtypes. The FDA approval of ICI plus chemotherapy in TNBC will provide real-world data that will help to better evaluate the benefit of this therapeutic strategy in underrepresented in landmark clinical trials populations, specifically Black patients. Comprehensive translational research and the use of biomarkers will help avoid the development of “add-on designs” which adds a new immune drug to a clinically established modality without leading to the development of adequate strategies for each individual patient. Indeed, the first results from biomarker analyses in immunotherapy TNBC trials highlight the heterogeneity of this disease and the urgent need to better characterize the TME to tailor immunotherapeutic approaches^37,86^. The predictive value of several biomarkers, including TIL levels, presence of tertiary lymphoid structures, or expression of immune gene signatures, is under investigation and has already been retrospectively evaluated in some clinical trials^7,37,87^. Only PD-L1 IHC expression is currently used to select TNBC patients for ICI in the metastatic setting. Moreover, its use in clinical practice remains controversial and complicated by the availability of several mAb and scoring systems and by the limited inter-observer agreement of PD-L1 scoring^88^. Blood-based biomarker research is ongoing, and liquid biopsies may become a noninvasive alternative to tissue biopsies in predicting and monitoring treatment responses.

Immunotherapy is associated with unique and sometimes severe irAEs that will require multidisciplinary collaborative efforts to offer adequate management of the increasing number of patients treated with ICI and to treat emerging toxicity from new immune-modulating agents and ACTs^82^. Another challenge for developing immunotherapy is to define an adequate response assessment, as the pattern of responses to ICI is different from that due to chemotherapeutic agents. Immune Response Evaluation Criteria in Solid Tumors (iRECIST) to better capture the benefit of immunotherapy have been developed, but most trials are still using the conventional RECIST^89^. In BC, pCR after NACT is a surrogate endpoint for a long-term clinical outcome, which might be less appropriate to capture long-term immune memory responses that could sustain therapeutic effects and prevent relapses, as recently suggested by the results of the GeparNUEVO study^32,38^. The development of adequate endpoints and new imaging techniques to measure the immune response could refine our approach to tumor response assessment and our criteria predictive of benefit from a given therapy.

Future clinical investigations will also need to address the question of de-escalation strategies for patients with long-term benefits. The excellent outcome observed in the absence of chemotherapy in patients with high TILs, and early-stage TNBC has led to the design of neoadjuvant immunotherapy trials omitting chemotherapy (e.g., NCT04427293)^90^. For non-responders, the improved understanding of tumor-immune interactions and the contribution of the TME, notably with the help of the latest technologies such as single-cell analyses and spatial transcriptomics, may provide new drug targets and strategies to overcome resistance^91,92^.

In summary, the clinical research landscape of immunotherapy in BC is expanding with novel investigational therapies aimed at initiating, restoring, or triggering patients’ immune responses against tumor cells. Innovative drugs combinations have already demonstrated an improved outcome for some BC patients, and these new therapeutic strategies will gradually be integrated into clinical treatments.

### Reporting summary

Further information on research design is available in the Nature Research Reporting Summary linked to this article.

## Supplementary information

Supplementary Material

Reporting Summary

## Data Availability

The data used for the Fig. 1 design are available in supplementary table 1. Data extracted from https://clinicaltrials.gov/ with research terms “breast”, “nivolumab”, “pembrolizumab”, “avelumab”, “atezolizumab”, “durvalumab”, “ipilimumab”, “tremelimumab”, “CAR-T”, “Bispecific”, “Vaccine”, “immunotherapy”, “4-1BB”, “OX-40”, “LAG”, “TIGIT”, “PD-1”, “PD-L1”, and “NK cells”. Data extracted on January 14, 2022.
